# Posterior Arytenoid Cartilage Dislocation Despite Optimal Intubation During Prolonged Steep Trendelenburg Robotic Prostatectomy: A Potential Biomechanical Contributor

**DOI:** 10.3390/jcm15072652

**Published:** 2026-03-31

**Authors:** Seong Hyeok Lee, Hyun Jung Koh

**Affiliations:** Department of Anesthesiology and Pain Medicine, Seoul St. Mary’s Hospital, College of Medicine, The Catholic University of Korea, 222 Banpo-daero, Seocho-gu, Seoul 06591, Republic of Korea; dtg1836@gmail.com

**Keywords:** arytenoid cartilage dislocation, postoperative hoarseness, robot-assisted radical prostatectomy (RARP), Trendelenburg position, vocal cord paralysis, video laryngoscopy

## Abstract

**Background**: Arytenoid cartilage dislocation (ACD) is a rare but clinically significant complication of endotracheal intubation that may be misdiagnosed as transient vocal cord paralysis. The potential role of prolonged surgical positioning in ACD remains underrecognized. **Methods**: A 63-year-old male developed left posterior ACD following robot-assisted radical prostatectomy (RARP) performed in a steep Trendelenburg position for 3.5 h. Intubation was successfully achieved on the first attempt using a video laryngoscope with full glottic visualization and no apparent airway trauma. Postoperatively, the patient developed persistent dysphonia, dysphagia, aspiration, and tongue deviation. Initial flexible laryngoscopy suggested left vocal cord paralysis, whereas direct laryngoscopy on postoperative day 6 confirmed posterior arytenoid dislocation. Urgent closed reduction was performed, followed by structured voice therapy, which resulted in substantial functional recovery. **Discussion**: This case illustrates that ACD may occur despite technically optimal and atraumatic intubation and should be interpreted as reflecting a temporal association rather than a definitive causal relationship. Prolonged steep Trendelenburg positioning and extended operative duration may be considered potential contributing biomechanical factors, possibly mediated by venous congestion, mucosal edema, altered endotracheal tube dynamics, and cumulative shear stress on the cricoarytenoid joint. However, these mechanisms remain interpretive and hypothesis-generating. **Conclusions**: Prolonged steep Trendelenburg positioning and extended operative duration may represent possible contributing biomechanical factors in ACD, even in the setting of atraumatic intubation. Early laryngeal evaluation and timely reduction remain essential for optimal functional recovery.

## 1. Introduction

Airway management via endotracheal intubation is a cornerstone of modern anesthesiology; however, it is not without infrequent yet debilitating complications. Current guidelines emphasize that comprehensive airway assessment should precede airway instrumentation to minimize perioperative airway-related risks [1]. Among these, arytenoid cartilage dislocation (ACD) is considered a rare complication of airway instrumentation. However, its true incidence is likely underestimated, as it is frequently misinterpreted as transient vocal fold paralysis or postoperative hoarseness [2,3,4]. Importantly, ACD represents a clinically significant cause of persistent postoperative hoarseness following endotracheal intubation, often leading to dysphonia, aspiration, and impaired airway protection when diagnosis is delayed [5,6]. Although the reported incidence ranges from 0.01% to 0.1%, underrecognition remains common due to frequent misinterpretation as transient laryngeal edema or vocal fold paralysis in the immediate postoperative period [2]. Consequently, the majority of published reports have focused on mechanically challenging airways or overt procedural trauma as the central etiologic mechanisms [7,8,9].

Emerging evidence, however, suggests that ACD may occur even in the absence of technically difficult intubation and that additional perioperative variables, such as intraoperative head and neck positioning, surgical duration, and endotracheal tube biomechanics, may contribute to its development [5,10,11]. In particular, procedures requiring prolonged steep Trendelenburg positioning, including robot-assisted pelvic surgeries, generate unique physiological and mechanical stresses within the upper airway that are not routinely considered in conventional airway risk assessment. Venous congestion, pharyngolaryngeal edema, altered intracuff pressure, and gravitational displacement of the endotracheal tube may collectively increase the vulnerability of the cricoarytenoid joint to injury.

Despite the expanding use of robotic surgery and prolonged Trendelenburg positioning across multiple surgical disciplines, the relationship between surgical posture and ACD remains poorly explored in the current literature. Most studies continue to conceptualize ACD primarily as a complication of the intubation maneuver itself, rather than as a multifactorial injury influenced by operative biomechanics.

In this context, we present a case of posterior arytenoid cartilage dislocation (ACD) following prolonged steep Trendelenburg robotic surgery and explore the hypothesis that surgical positioning may act as a contributing biomechanical factor, rather than implying a direct causal relationship. Beyond the clinical description, this case serves as a framework to explore potential biomechanical mechanisms and to contextualize emerging evidence regarding non-traumatic contributors to ACD. By integrating this clinical presentation with current evidence on airway biomechanics, we aim to highlight prolonged Trendelenburg positioning as a potential contributor to ACD, emphasize the diagnostic pitfalls that may delay appropriate management, and propose heightened vigilance strategies for contemporary anesthetic practice.

This report is not intended as a standalone case description but rather as a case-based analytical discussion that integrates clinical observation with existing evidence to explore potential non-traumatic mechanisms of arytenoid cartilage dislocation, while acknowledging the limitations of a single-case framework.

## 2. Materials and Methods

A 63-year-old man (186 cm, 72 kg) without medical comorbidities underwent robot-assisted radical prostatectomy for incidentally detected prostate cancer. His occupation required extensive voice use, and although he frequently experienced vocal fatigue, he had no history of dysphonia or swallowing difficulty.

General anesthesia was induced and maintained by an experienced board-certified anesthesiologist. Endotracheal intubation was performed smoothly on the first attempt using a video laryngoscope with a full view of the vocal folds (Cormack–Lehane grade I). An 8.0 mm internal diameter endotracheal tube was advanced beyond the vocal cords and secured at 23 cm at the right lip corner. Anesthesia was maintained with oxygen, air, and desflurane. The cuff was inflated with approximately 7 mL of air. Initial cuff pressure measured using a manometer was within 20–25 cm H_2_O, and clinical assessment suggested adequate cuff inflation without evidence of overinflation. The initial peak airway pressure (P_aw_) was approximately 15 cm H_2_O and remained within 22–24 cm H_2_O throughout the operation during steep Trendelenburg position. The total operative time was approximately 3.5 h, during which a steep Trendelenburg position was continuously maintained. Extubation was uneventful without excessive coughing or agitation. No intraoperative head or neck repositioning was performed, and no oral adjunct devices (e.g., bite block) were used.

## 3. Results

Immediately after the operation, the patient developed aphonia, dysphagia, and decreased tongue mobility accompanied by hypoesthesia on the left side. These symptoms were initially attributed to transient intubation-related neuropraxia. Oral intake was restricted on the operative day. However, on postoperative day 1, attempts at a liquid diet resulted in repeated aspiration, persistent hoarseness and ongoing swallowing difficulty. Notably, brief improvement in phonation occurred during respiratory exercises at rest.

Due to a lack of improvement, otolaryngologic evaluation was obtained on postoperative day 3. Flexible laryngoscopy demonstrated left vocal fold immobility in a paramedian position with mild ipsilateral tongue swelling and restricted movement. Conservative observation was initially recommended based on the presumed diagnosis of vocal fold paralysis in the early postoperative period.

Despite ongoing monitoring, symptoms worsened, with progressive swallowing difficulty even for liquids by postoperative day 6. Although initial laryngoscopic evaluation suggested vocal fold immobility, repeat examination revealed posterior dislocation of the left arytenoid cartilage. Additional diagnostic modalities such as computed tomography or laryngeal electromyography were not performed. The diagnosis was established based on direct laryngoscopic findings and was further supported by immediate anatomical and functional improvement following closed reduction. This clinical course highlights the potential difficulty in distinguishing arytenoid dislocation from vocal fold paralysis in the early postoperative period. Emergency closed mechanical reduction was performed the following day, achieving immediate anatomical restoration with prompt clinical improvement, further lending support to the diagnosis. Swallowing function improved promptly; however, dysphonia persisted. The patient subsequently underwent structured voice therapy for two months, leading to marked recovery of vocal function.

**Figure 1:**
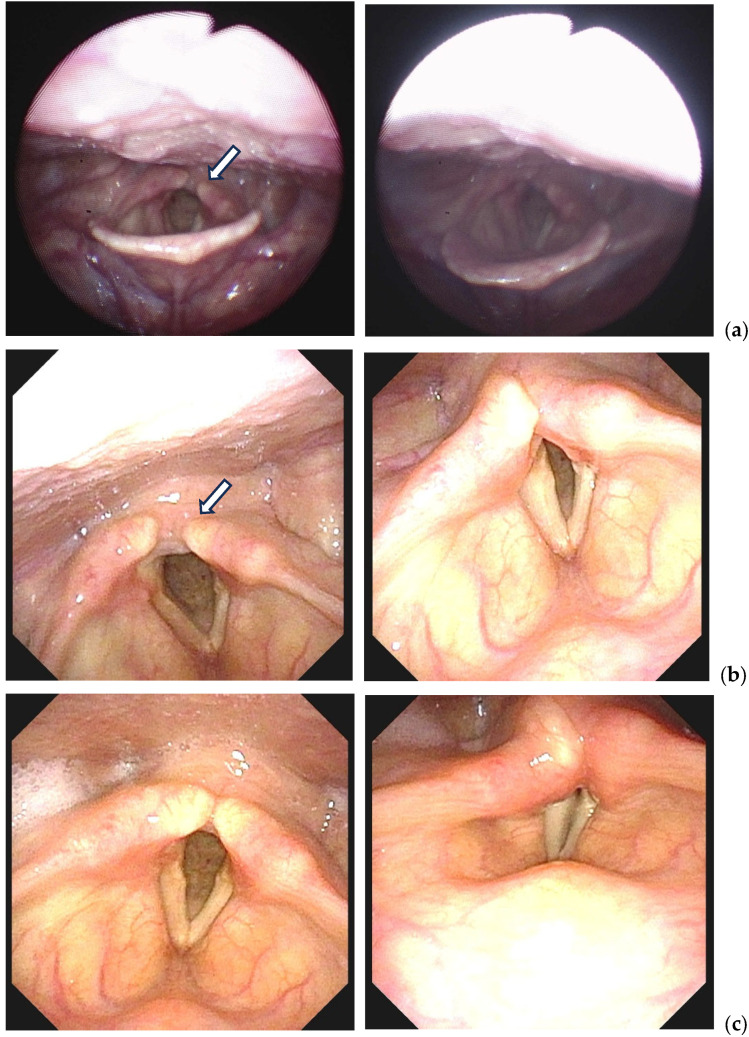
Serial laryngoscopic findings of left posterior arytenoid cartilage dislocation before and after surgical reduction. (**a**) Initial laryngoscopic examination performed on postoperative day 3 revealed posterior displacement of the left arytenoid cartilage with vocal fold immobility in a paramedian position and asymmetry of the glottic aperture. (**b**) Repeat laryngoscopic findings on postoperative day 6, prior to arytenoid reduction, showed persistent posterior dislocation with progressive glottic narrowing and incomplete vocal fold closure. (**c**) Laryngoscopic findings obtained 2 days after arytenoid reduction demonstrated restoration of arytenoid position, improved glottic symmetry, and normalized vocal fold movement. arrow: dislocated left arytenoid cartilage.

This case highlights delayed recognition of posterior arytenoid cartilage dislocation presenting as combined vocal fold immobility and hypoglossal nerve-related symptoms following uneventful intubation in prolonged robotic surgery with steep Trendelenburg positioning. Early consideration of arytenoid dislocation in patients with postoperative dysphonia is essential, as timely mechanical reduction combined with voice therapy appears critical for optimal functional recovery (Figure 1).

## 4. Discussion

ACD is a mechanical disruption of the cricoarytenoid joint and remains an underrecognized cause of persistent postoperative hoarseness following endotracheal intubation [8,12,13]. This report is not intended as a standalone case description but rather as a case-based analytical communication that integrates clinical observation with existing evidence to explore potential non-traumatic mechanisms of ACD. Clinically, it presents with dysphonia, vocal fatigue, dysphagia, aspiration, anterior neck discomfort, and occasionally airway compromise [9,14]. Because vocal fold immobility is commonly observed on laryngoscopy, ACD is frequently misdiagnosed as recurrent laryngeal nerve paralysis or postoperative edema, leading to delayed definitive management and suboptimal functional recovery [2,15,16,17].

Persistent hoarseness lasting beyond several postoperative days—particularly after resolution of routine sore throat—should prompt formal laryngeal evaluation [18,19]. Flexible laryngoscopy allows assessment of arytenoid position and vocal fold mobility, while Strobovideolaryngoscopy assists in distinguishing mechanical fixation from neurogenic paralysis [8]. When findings remain equivocal, focused laryngeal computed tomography can provide structural confirmation of joint displacement [10]. Early closed reduction is the main therapeutic intervention and offers the greatest likelihood of complete recovery when performed promptly [20]. Delayed recognition may permit fibrotic remodeling and progressive joint fixation, necessitating more invasive surgical procedures and prolonged voice rehabilitation [15].

Traditionally, ACD has been attributed to traumatic airway manipulation, excessive laryngoscopic force, rigid stylet use, difficult intubation, prolonged intubation, forceful extubation, or vigorous coughing. Accordingly, most published reports conceptualize ACD as an intubation event-related injury, in which technical difficulty or overt airway trauma serves as the principal precipitating events [5,9,11,12,21].

In contrast, the present case challenges this conventional model. Intubation was accomplished on the first attempt using video laryngoscopy with full glottic visualization, minimal lifting force, stable ventilation parameters, and atraumatic extubation performed by an experienced anesthesiologist. These features make significant procedural trauma less likely; however, subtle intubation- or extubation-related mechanical injury cannot be completely excluded. Instead, this case may suggest a possible alternative pathophysiological framework in which prolonged steep Trendelenburg positioning and operative duration may act as biomechanical stressors, potentially capable of contributing to destabilizing the cricoarytenoid joint despite technically optimal airway management.

### 4.1. Role of Prolonged Trendelenburg Positioning as a Biomechanical Stressor

The most distinctive feature of this case is the maintenance of steep Trendelenburg positioning for more than 3.5 h during robot-assisted pelvic surgery. Unlike conventional supine procedures, prolonged head-down tilt has been associated with physiological and mechanical alterations in the upper airway, including upper airway edema and increased airway resistance [22,23].

First, venous congestion and impaired lymphatic drainage promote pharyngolaryngeal edema [24,25]. Edematous tissues may exhibit reduced structural resilience, potentially lowering the threshold for arytenoid displacement.

Second, positional changes combined with pneumoperitoneum can alter endotracheal tube orientation and intracuff pressure dynamics [26,27,28]. Even without visible tube migration, subtle cephalad displacement or focal pressure concentration could theoretically occur.

Third, extended operative duration allows repetitive micromovement of the endotracheal tube with ventilation cycles and minor patient adjustments. While individually minimal, these repetitive shear forces may accumulate and contribute to a strain-type injury to the cricoarytenoid joint [29], although this remains speculative.

Collectively, these considerations raise the possibility that steep Trendelenburg positioning may not be entirely passive and could act as a potential biomechanical amplifier of laryngeal stress. Within this framework, joint destabilization may arise from cumulative load exposure rather than from a single traumatic event. These proposed mechanisms should be interpreted as hypothesis-generating and require further investigation.

In addition to dysphonia and dysphagia, this patient also demonstrated tongue deviation, reduced tongue mobility, and unilateral hypoesthesia, which are not fully explained by isolated arytenoid dislocation. These findings raise the possibility of concomitant cranial nerve involvement, particularly hypoglossal neuropraxia or compressive neuropathy. In this case, these neurological findings were unexpected, as the intubation was atraumatic, no airway adjuncts were used, and the surgical procedure did not involve direct manipulation of the head and neck region. One possible explanation is transient compressive neuropathy related to intraoperative positioning. In the setting of prolonged steep Trendelenburg positioning, the tongue may have been displaced and subjected to sustained pressure between surrounding structures, potentially exacerbated by perioperative dehydration. However, this mechanism remains speculative, as no direct intraoperative evidence of such compression was documented. The gradual recovery of tongue movement and sensory function over time suggests a transient and reversible process, consistent with neuropraxia rather than permanent nerve injury. Therefore, while arytenoid dislocation was considered the primary cause of dysphonia, the possibility of concomitant transient neuropathic involvement cannot be excluded.

However, current evidence regarding the mechanisms of arytenoid dislocation in the setting of non-difficult intubation and robotic surgery remains limited and inconclusive. While several biomechanical and positional factors have been proposed, direct causal relationships have not been established, and most available data are derived from isolated case reports or small case series. Therefore, these proposed mechanisms should be interpreted with caution, and further systematic investigation is required to clarify their clinical significance.

### 4.2. Reconsidering the Role of Video Laryngoscopy in Arytenoid Vulnerability

Video laryngoscopy is generally associated with reduced airway trauma; however, blade mechanics and lifting vectors remain relevant. Direct epiglottic evaluation, particularly when force is concentrated at the epiglottic tip rather than indirectly via the vallecula, may transiently narrow the hypopharyngeal space and increase localized stress on the arytenoid structures [30,31].

In right-handed operators, it may be hypothesized that lifting vectors may preferentially transmit force toward the left arytenoid, potentially contributing to the reported predominance of left-sided ACD [2]; this remains speculative and has not been directly demonstrated. In the present case, although no overt trauma was observed, subtle mechanical stress during laryngoscopy may have rendered the joint susceptible, with subsequent prolonged Trendelenburg positioning acting as a secondary amplifying factor. This interaction may be consistent with a multifactorial “two-hit” model rather than a purely intubation-driven injury, and should be interpreted as a hypothesis-generating concept rather than an established mechanism. In summary, the proposed “two-hit” framework suggests that atraumatic intubation may create a vulnerable laryngeal condition, while prolonged surgical positioning and operative duration may act as secondary contributing biomechanical factors, although this interpretation remains hypothetical.

### 4.3. Diagnostic Delay and Its Clinical Implications

This case also illustrates the diagnostic challenge of distinguishing ACD from vocal fold paralysis. Additional diagnostic modalities such as computed tomography or laryngeal electromyography may be useful in selected cases when the diagnosis is uncertain. However, in the present case, the diagnosis was established based on repeat laryngoscopic findings and was further supported by immediate anatomical and functional improvement following closed reduction. These findings highlight that early laryngoscopic evaluation may not always be definitive and that repeat assessment can be critical for accurate diagnosis. Initial laryngoscopy demonstrated vocal fold immobility without clear recognition of arytenoid malposition, leading to conservative observation.

Evidence indicates that early closed reduction—ideally within days of injury—yields superior phonatory and swallowing outcomes compared with delayed intervention [16,32,33,34]. Rapid fibrotic change and progressive joint fixation may transform a reversible mechanical dislocation into chronic structural dysfunction. Although reduction ultimately resulted in substantial recovery in this patient, the delay underscores the importance of maintaining structural suspicion in high-risk surgical contexts.

In addition, the timing of specialist evaluation may be influenced by real-world clinical workflow constraints, including the prioritization of urgent cases and the availability of subspecialty assessment. Such factors may contribute to delays in definitive evaluation in patients with initially non–life-threatening symptoms.

### 4.4. Clinical Implications in Contemporary Robotic Surgery

As robotic pelvic surgeries expand across urology, gynecology, and colorectal disciplines, prolonged steep Trendelenburg positioning has become routine. Nevertheless, airway risk assessment remains predominantly focused on intubation difficulty rather than on positional biomechanics.

This case may suggest a conceptual shift in understanding ACD as a multifactorial injury influenced by surgical positioning, operative duration, airway biomechanics, rather than solely an intubation-related complication. Recognition of these factors may inform potential preventive considerations. However, as these variables were not directly measured or shown to be abnormal in this case, such recommendations should be interpreted as prudent extrapolations rather than case-proven preventive measures. These may include careful cuff pressure monitoring, periodic reassessment of endotracheal tube position during prolonged head-down procedures, minimization of unnecessary laryngoscopic force, and early postoperative voice evaluation in high-risk settings.

Importantly, the principal message of this report is that prolonged steep Trendelenburg positioning and operative duration may represent possible contributing biomechanical factors in the development of posterior ACD, even in the setting of atraumatic and technically optimal intubation, rather than representing a primary causal mechanism. Early recognition and timely reduction remain essential to optimize functional recovery.

From a practical perspective, several preventive and early detection strategies may be considered. Careful monitoring of endotracheal tube cuff pressure, in accordance with current airway management guidelines [1], may help reduce excessive mechanical stress on laryngeal structures. In addition, periodic reassessment of endotracheal tube position during prolonged procedures—particularly in steep Trendelenburg positioning—may be beneficial. Finally, early otolaryngologic evaluation should be considered in patients with persistent postoperative dysphonia to facilitate timely diagnosis and intervention.

Importantly, several factors relevant to the proposed mechanisms were not directly measured in this case. Serial cuff pressure trends were not recorded, objective assessment of postoperative airway edema was not performed, and no radiologic or dynamic evidence of endotracheal tube migration was available. Therefore, while arytenoid dislocation was considered the primary cause of dysphonia, the observed neurological findings were considered transient and likely secondary, and were not regarded as the primary mechanism underlying the patient’s dysphonia.

## 5. Conclusions

Posterior arytenoid cartilage dislocation may occur despite atraumatic, technically optimal intubation during prolonged steep Trendelenburg robotic surgery. This case is consistent with the interpretation that surgical positioning may act as a potential contributing biomechanical factor, particularly in the absence of direct physiological or mechanical measurements in this case.

Importantly, this case highlights a potentially underrecognized clinical scenario and underscores the need for heightened clinical awareness. Early diagnosis and timely reduction are essential for optimal functional recovery. In addition, transient neurological findings observed in this case were considered secondary and not central to the primary structural diagnosis. These findings may be interpreted within a hypothesis-generating “two-hit” framework.

## Data Availability

The data supporting the findings of this study are contained within the article.

## References

[1] Gómez-Ríos M., Sastre J.A., Onrubia-Fuertes X., López T., Abad-Gurumeta A., Casans-Frances R., Gómez-Ríos D., Garzón J.C., Martínez-Pons V., Casalderrey-Rivas M. (2024). Executive Summary of the Spanish Society of Anesthesiology, Reanimation and Pain Therapy (SEDAR) Spanish Society of Emergency and Emergency Medicine (SEMES) and Spanish Society of Otolaryngology, Head and Neck Surgery (SEORL-CCC) Guideline for difficult airway management. Acta Otorrinolaringol. Esp..

[2] Chun E.H., Baik H.J., Chung R.K., Lee H.J., Shin K., Woo J.H. (2017). Arytenoid cartilage dislocation mimicking bilateral vocal cord paralysis: A case report. Medicine.

[3] Yan W.Q., Li C., Chen Z. (2022). Delayed diagnosis of arytenoid cartilage dislocation after tracheal intubation in the intensive care unit: A case report. World J. Clin. Cases.

[4] Goz V., Qureshi S., Hecht A.C. (2013). Arytenoid dislocation as a cause of prolonged hoarseness after cervical discectomy and fusion. Glob. Spine J..

[5] Tsuru S., Wakimoto M., Iritakenishi T., Ogawa M., Hayashi Y. (2017). Cardiovascular operation: A significant risk factor of arytenoid cartilage dislocation/subluxation after anesthesia. Ann. Card. Anaesth..

[6] Senoglu N., Oksuz H., Ugur N., Dogan Z., Kahraman A. (2008). Arytenoid dislocation related to an uneventful endotracheal intubation: A case report. Cases J..

[7] Rubin A.D., Hawkshaw M.J., Moyer C.A., Dean C.M., Sataloff R.T. (2005). Arytenoid Cartilage Dislocation: A 20-year Experience. J. Voice.

[8] Lombardi R.A., Arthur M.E. (2025). Arytenoid Subluxation. StatPearls.

[9] Fujii-Abe K., Ikeda M., Yajima M., Kawahara H. (2023). A Case of Anterior Arytenoid Cartilage Dislocation During Nasal Tracheal Intubation Using an Indirect Video Laryngoscope. Anesth. Prog..

[10] Alalyani N.S., Alhedaithy A.A., Alshammari H.K., AlHajress R.I., Alelyani R.H., Alshammari M.F., Alhalafi A.H., Alharbi A., Aldabal N. (2024). Incidence and Risk Factors of Arytenoid Dislocation Following Endotracheal Intubation: A Systematic Review and Meta-Analysis. Cureus.

[11] Kong X., Song Y., Wang L., He G., Ma C., Zhao R., Wang M., Shi L., Cui W. (2022). Risk factors of arytenoid dislocation after endotracheal intubation: A propensity-matched analysis. Laryngoscope Investig. Otolaryngol..

[12] Oh T.K., Yun J.Y., Ryu C.H., Park Y.N., Kim N.W. (2016). Arytenoid dislocation after uneventful endotracheal intubation: A case report. Korean J. Anesthesiol..

[13] Kiran S., Tandon U., Dwivedi D., Kumar R. (2016). Prolonged hoarseness following endotracheal intubation―not so uncommon?. Indian J. Anaesth..

[14] Lee Y., Park H., Park J.E., Kim S.K., Park E.S., Rha D.W. (2020). Incidental Diagnosis of Pediatric Arytenoid Cartilage Dislocation During Videofluoroscopic Swallowing Study: A Case Report. Ann. Rehabil. Med..

[15] Sataloff R.T. (1998). Arytenoid dislocation: Techniques of surgical reduction. Oper. Tech. Otolaryngol. Head Neck Surg..

[16] Lee S.W., Park K.N., Welham N.V. (2014). Clinical features and surgical outcomes following closed reduction of arytenoid dislocation. JAMA Otolaryngol. Head Neck Surg..

[17] Zhuang P., Nemcek S., Surender K., Hoffman M.R., Zhang F., Chapin W.J., Jiang J.J. (2013). Differentiating arytenoid dislocation and recurrent laryngeal nerve paralysis by arytenoid movement in laryngoscopic video. Otolaryngol. Head Neck Surg..

[18] Teng Y., Wang H.E., Lin Z. (2014). Arytenoid cartilage dislocation from external blunt laryngeal trauma: Evaluation and therapy without laryngeal electromyography. Med. Sci. Monit..

[19] Yamanaka H., Hayashi Y., Watanabe Y., Uematu H., Mashimo T. (2009). Prolonged hoarseness and arytenoid cartilage dislocation after tracheal intubation. Br. J. Anaesth..

[20] Li X., Wang L., Lou Z., Lin Z. (2024). Bilateral arytenoid dislocation after an emergency tracheal intubation: A rare case report. Acta Oto-Laryngol. Case Rep..

[21] Pacheco-Lopez P.C., Berkow L.C., Hillel A.T., Akst L.M. (2014). Complications of Airway Management. Respir. Care.

[22] Kilic O.F., Börgers A., Köhne W., Musch M., Kröpfl D., Groeben H. (2014). Effects of steep Trendelenburg position for robotic-assisted prostatectomies on intra- and extrathoracic airways in patients with or without chronic obstructive pulmonary disease. Br. J. Anaesth..

[23] Rajmohan N., Panikkaparambil S., Ramkumar P., Nair S.G., Shaji A., Menon L.P. (2026). Observational study of airway changes in robotic-assisted laparoscopic surgeries in the steep Trendelenburg position after a short period of post-operative ventilation. J. Minim. Access Surg..

[24] Vaithialingam B., Masapu D., Rudrappa S. (2022). Massive Congestive Facial and Submandibular Oedema Due to Extreme Neck Flexion Following Suboccipital Craniectomy: A Case Report. Cureus.

[25] Henderson A.C., Levin D.L., Hopkins S.R., Olfert I.M., Buxton R.B., Prisk G.K. (2006). Steep head-down tilt has persisting effects on the distribution of pulmonary blood flow. J. Appl. Physiol..

[26] Alvarez M., Llanes Rico S., Tsai J., Schaffer R.M., Masri M., Sciarra J., Kuchciak A. (2021). Endotracheal Tube Migration in Steep Trendelenburg Position with the Estape TrenMAX Positioning System. Cureus.

[27] Kwon Y., Jang J.S., Hwang S.M., Lee J.J., Hong S.J., Hong S.J., Kang B.Y., Lee H.S. (2019). The Change of Endotracheal Tube Cuff Pressure During Laparoscopic Surgery. Open Med..

[28] Inada T., Uesugi F., Kawachi S., Takubo K. (1996). Changes in tracheal tube position during laparoscopic cholecystectomy. Anaesthesia.

[29] Friedman A.D., Kobler J.B., Landau-Zemer T., Barbu A.M., Burns J.A. (2012). High-force simulated intubation fails to dislocate cricoarytenoid joint in ex vivo human larynges. Ann. Otol. Rhinol. Laryngol..

[30] Oh J.Y., Lee J.H., Kim Y.Y., Baek S.M., Jung D.W., Park J.H. (2021). A comparative study of glottis visualization according to the method of lifting the epiglottis in video laryngoscopy: Indirect and direct lifting methods. Anesth. Pain Med..

[31] Choi S., Lee D.J., Shin K.W., Kim Y.J., Park H.P., Oh H. (2023). Direct versus indirect epiglottis elevation in cervical spine movement during videolaryngoscopic intubation under manual in-line stabilization: A randomized controlled trial. BMC Anesthesiol..

[32] Frosolini A., Caragli V., Badin G., Franz L., Bartolotta P., Lovato A., Vedovelli L., Genovese E., de Filippis C., Marioni G. (2025). Optimal Timing and Treatment Modalities of Arytenoid Dislocation and Subluxation: A Meta-Analysis. Medicina.

[33] Lee D.H., Yoon T.M., Lee J.K., Lim S.C. (2013). Treatment outcomes of closed reduction of arytenoid dislocation. Acta Otolaryngol..

[34] Liu K., Hu H., Lu Y., Yu Z. (2024). A Novel Five-Step Reduction Technique of Arytenoid Dislocation. Laryngoscope.

